# Severe Ovarian Hyperstimulation Syndrome in the Setting of In Vitro Fertilization Treatment

**DOI:** 10.7759/cureus.39939

**Published:** 2023-06-04

**Authors:** Hannia Diaz Ayllon, Oscar L Hernandez, Talwinder Nagi, Carlos M Cespedes

**Affiliations:** 1 Internal Medicine, Florida Atlantic University Charles E. Schmidt College of Medicine, Boca Raton, USA; 2 Medicine, Bethesda Hospital East, Boynton Beach, USA

**Keywords:** ovarian follicle, oocyte retrieval, ovarian hyperstimulation, large pleural effusion, massive ascites

## Abstract

Ovarian hyperstimulation syndrome (OHSS) is one of the complications of pharmacological ovarian stimulation used in fertility treatments. This syndrome is characterized by increased vascular permeability secondary to stimulation, resulting in a fluid shift from the intravascular space to the third-space compartments. Patients developing OHSS can experience severe complications, including ascites, pleural effusions, and shock. Here, we present a case of OHSS in the setting of recent transvaginal oocyte retrieval, leading to severe ascites, pleural effusion, and hypotension requiring urgent intervention.

## Introduction

In vitro fertilization (IVF), viewed as the gold standard in fertility treatment, has become a widely used form of assisted reproductive technology (ART) [1]. While generally safe, the pharmacological ovarian stimulation used for IVF poses a risk for significant complications, the most serious being ovarian hyperstimulation syndrome (OHSS). While the exact mechanism behind this syndrome is obscure, it is characterized by cystic enlargement of the ovaries and a fluid shift from the intravascular space into the interstitial space due to increased capillary permeability. Clinical presentation can vary in severity from mild forms with abdominal discomfort and nausea to severe, life-threatening complications. Mild OHSS is defined by symptoms, including nausea, vomiting, and abdominal distention. While these symptoms, in conjunction with ultrasonographic evidence of ascites, define moderate OHSS. Severe OHSS is reserved for cases that include hypotension, dyspnea, or evidence of reduced renal perfusion [2]. The incidence of moderate to severe OHSS is estimated to be approximately 1%-5% in all IVF cycles, with an associated mortality of roughly one in 50,000 individuals [2,3]. With an increase in the number of patients opting for ART, physicians must be aware of this rare clinical condition and its presentations, including multi-organ dysfunction and death. We present a case of severe OHSS in a patient following ovarian stimulation therapy for transvaginal oocyte retrieval, leading to large-volume ascites and symptomatic pleural effusions.

## Case presentation

A 28-year-old female with no previous medical history presented to the emergency department (ED) with a chief complaint of chest pain and exertional dyspnea. Her symptoms began approximately seven days prior, for which she initially sought an ED evaluation at another facility five days prior to her presentation. At that time, she complained of nausea and non-bloody, non-bilious emesis. She was given supportive care with intravenous (IV) fluid and antiemetics, after which she was subsequently discharged from the ED for further outpatient follow-up. During her presentation to our medical facility, she complained of similar nausea and vomiting in addition to severe dyspnea and abdominal distention. Her shortness of breath occurred with exertion and was associated with orthopnea. She denied any fevers, chills, or productive coughs. Furthermore, she denied any prior pregnancies and reported regular menses with her last menstrual period, reportedly 23 days before presentation. A urine pregnancy test was negative.

**Figure 1:**
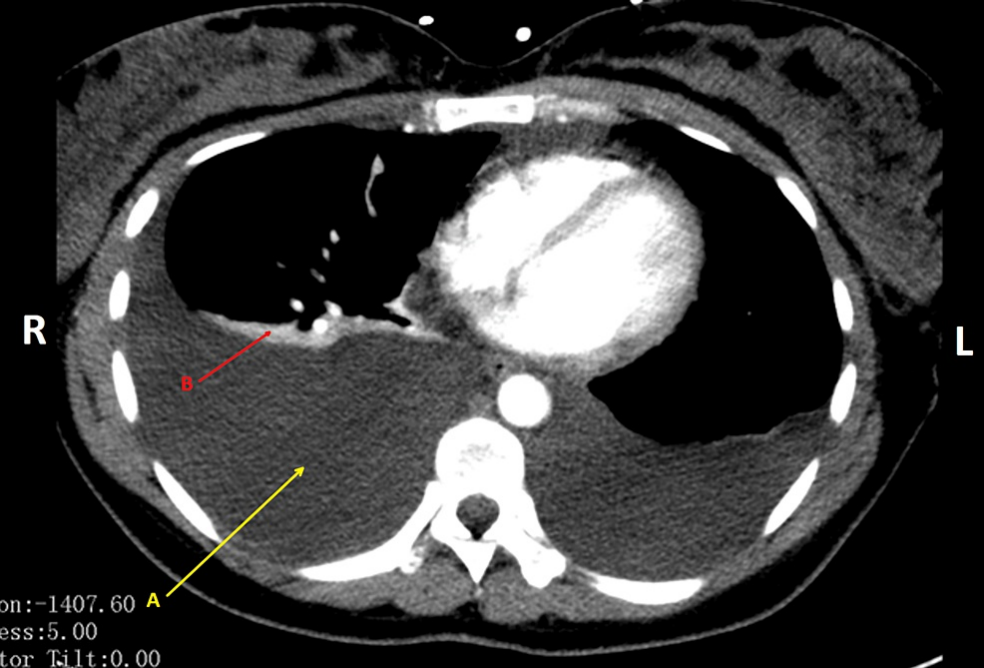
CT imaging of the chest demonstrated a large right pleural effusion and a moderate left pleural effusion. Note the moderate right pleural effusion (A) with associated atelectasis (B).

**Figure 2:**
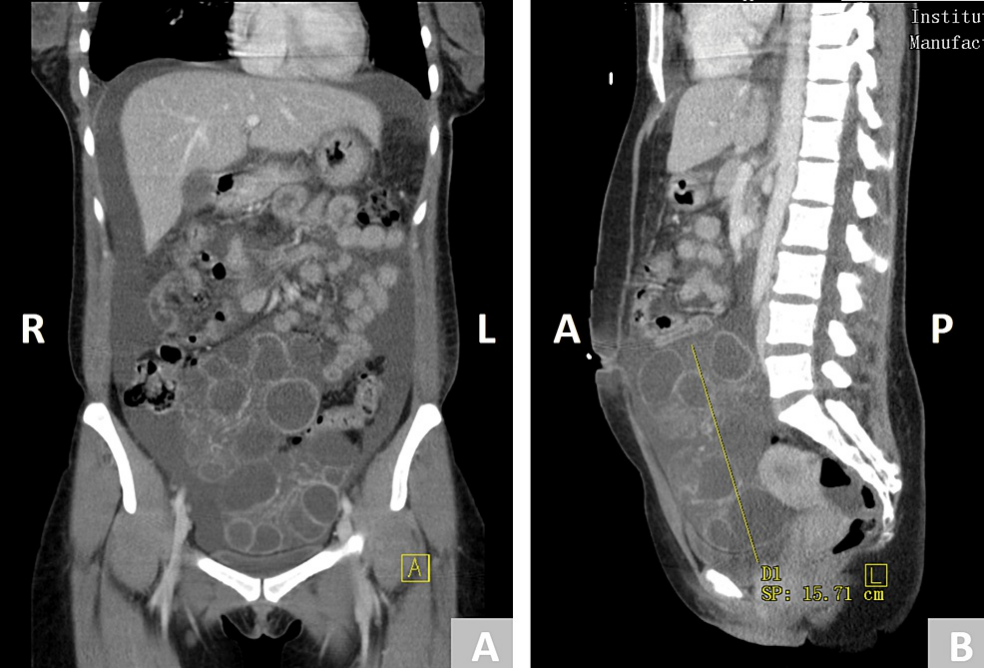
Coronal (2A) and sagittal (2B) views of CT imaging of the abdomen and pelvis demonstrate ascites with a large collection of ovarian follicles.

In the ED, the patient was tachycardic with a heart rate of 130 beats per minute (BPM) and hypotensive with an initial blood pressure of 90/67 mmHg. Physical examination revealed bilateral decreased breath sounds at the bases with dullness to percussion and a non-tender but distended abdomen without an appreciable fluid wave. The laboratory results obtained in the emergency department are summarized in Table 1. Initial laboratory results were significant for leukocytosis, elevated D-dimer, and a negative urine pregnancy test. Due to her labored breathing at presentation, an arterial blood gas (ABG) was drawn and showed a compensated metabolic acidosis. A computerized tomographic angiogram (CTA) of the chest, abdomen, and pelvis was conducted in order to evaluate her shortness of breath and abdominal distention. No evidence of a pulmonary embolism was seen; however, notable findings were discovered, including bilateral moderate pleural effusions with associated atelectasis (Figure 1), large-volume ascites, and large multicystic masses of the pelvis (Figures 2A, 2B). The patient was admitted for further evaluation of her intra-abdominal masses, ascites, and pleural effusions.

**Table 1:** Laboratory values on admission with comparable reference ranges

Laboratory Tests	Laboratory Values	Reference Range
Hematology		
Leukocyte Count	17,300/m^3^	4,500 – 11,000/m^3^
Hemoglobin	14.9 g/dL	12.1 - 15.1 g/dL
Hematocrit	44.0%	36.0 – 48.0%
Platelet Count	328,000/mm^3^	150,000 - 400,000/mm^3^
D-Dimer	8.65 μ/mL	< 0.4 μ/mL
Sedimentation Rate	16 mm/hr	0 – 20 mm/hr
Chemistry		
Sodium	130 mmol/L	136 – 145 mmol/L
Potassium	4.0 mmol/L	3.5 - 5.1 mmol/L
Chloride	99 mmol/L	101 – 111 mmol/L
Bicarbonate	17 mmol/L	21 – 32 mmol/L
Blood Urea Nitrogen	16 mg/dL	8 – 26 mg/dL
Creatinine	0.97 mg/dL	0.44 – 1.00 mg/dL
Glucose	95 mg/dL	65 – 100 mg/dL
C-Reactive Protein	2.223 mg/dL	0.000 - 0.748 mg/dL
Arterial Blood Gas		
pH	7.39	7.35-7.45
P_CO2_	30 mmHg	35 - 45 mmHg
P_O2_	93 mmHg	75 - 100mmHg
HCO3^-^	18.2 mmol/L	22-26 mmol/L

On admission day one, her preliminary ovarian and endometrial tumor marker results returned negative for cancer antigen 125 (CA 125) and human epididymis protein gene 4 (HE 4). Upon further discussion, the patient disclosed, in the absence of family members, that she had recently undergone infertility treatment one week prior. She reportedly received ovarian stimulation therapy with a multidrug regimen, including leuprolide, ganirelix, follitropin beta, menotropins, and urine-derived human chorionic gonadotropin the month prior, along with transvaginal oocyte retrieval one week before her initial presenting symptoms. Thus, our differential for a severe presentation of ovarian hyperstimulation syndrome (OHSS) was considered. Diagnostic and therapeutic paracentesis was offered for the patient’s ascites; however, she declined paracentesis and elected for less invasive interventions. Diuresis with IV furosemide 40 mg was conducted to reduce the ascites burden and her pleural effusions. Due to her tachycardia upon presentation and low blood pressure, she appeared to have intravascular depletion, presumably from her substantial fluid loss via her ascites, and was therefore also placed on IV fluids for intravascular repletion. Close monitoring of her urine output and her volume status were the initial steps of management. Throughout her hospital admission, she was given IV diuresis and supported with IV fluid hydration to balance the need for the reduction of her intraperitoneal volume overload while maintaining her intravascular volume. After two days, she experienced symptomatic relief and was deemed stable for discharge home with a five-day course of oral furosemide and close outpatient follow-up with her gynecologist and fertility specialist.

## Discussion

OHSS is a serious complication that carries significant morbidity and mortality. It is crucial for clinicians to identify and treat this syndrome early in its presentation to prevent further complications such as shock and death. While early identification and treatment can improve patient outcomes, early symptoms are often nonspecific, leading to delays in diagnosis and management. Thus, it is imperative for reproductive endocrinologists to educate patients on the warning signs of OHSS and for other clinicians to consider OHSS in their differential diagnosis, especially with the increased use of ART. Establishing a close physician-patient alliance in order to elicit essential information when considering a diagnosis of OHSS. In doing so, physicians should make efforts to ensure patient privacy and conduct interviews in a manner that is patient-centered and encourages the disclosure of sensitive information. It is important to be aware of high-risk patients since they require careful follow-up. These include younger age, polycystic ovarian syndrome, low BMI, rapidly rising serum estradiol levels, elevated peak estradiol levels, and a history of OHSS [4,5]. 

While the pathogenesis is poorly understood, vasoactive particles, including cytokines, tumor necrosis factor-α, endothelin-1, the renin-angiotensin system, and vascular endothelial growth factor (VEGF), have been implicated [6-8]. These cytokines have been associated with increased vascular permeability, especially within the peritoneal vasculature. Increased vascular permeability leads to the ascites noted in OHSS and can extend to the thoracic cavity through the semipermeable diaphragm if the ascites are large enough in volume. As such, pleural effusions are often associated with large-volume ascites. Ovarian cystic enlargement in response to stimulation causes initial mild symptoms of abdominal discomfort, nausea, and vomiting [2,9]. With increased vascular permeability, third-space fluid shift and intravascular volume depletion subsequently escalate the severity. In moderate cases, mild symptoms are accompanied by ultrasound-detected ascites due to third spacing and rupture of follicles [2]. Severe cases are characterized by clinically apparent ascites with dyspnea and/or pleural effusions due to fluid shift from abdominal ascites. Enlarged ovaries and ascites can restrict diaphragmatic movement, causing dyspnea even in the absence of effusions [2]. With increased intravascular depletion, OHSS can progress to life-threatening complications like hemoconcentration with thromboembolism, oliguria, pericardial effusions, and hypoxic respiratory distress. 

Mild cases are relatively common and seen in up to 5% of women undergoing IVF, while severe cases occur in only 0.5% of IVF cycles [9]. Persistent symptoms and progression to severe OHSS are more commonly observed in conception cycles [10]. However, rarer is the occurrence of severe OHSS in the absence of pregnancy, as seen in our case [10]. Due to the uncommon nature of this condition, there are minimal guidelines for categorizing and managing the disease. The Royal College of Obstetricians and Gynecologists proposed a classification system in 2016 that may be helpful for clinicians to reference when suspecting this condition, but a globally accepted algorithm for management is still not in place [6, 11]. OHSS is typically self-limiting; thus, treatment is mainly supportive. Management strategies often rely on fluid balance control, thromboembolic prophylaxis, and paracentesis with thoracentesis for large ascites and effusions. Techniques to increase intravascular oncotic pressure and reduce third spacing using IV albumin followed by diuresis are plausible treatments for patients declining invasive measures. Primary prevention strategies have been studied and deemed effective; however, more research is needed to clarify the pathophysiology behind the condition and significantly affect management [11,12].

## Conclusions

In patients receiving fertility treatments, severe ovarian hyperstimulation syndrome can occur, presenting as large-volume ascites, pleural effusions, and hypotension. This case represents a severe presentation of ovarian hyperstimulation syndrome requiring hospitalization. Swift recognition of this complication, followed by volume management, is essential for treating this potentially life-threatening complication. Detection and management of patients at risk of OHSS are of utmost importance to prevent progression to its severe form. As in our case, volume management may prove difficult for patients who decline invasive interventions.

## References

[1] Man A, Schwarz Y, Greif J (1997). Pleural effusion as a presenting symptom of ovarian hyperstimulation syndrome. Eur Respir J.

[2] Kumar P, Sait SF, Sharma A, Kumar M (2011). Ovarian hyperstimulation syndrome. J Hum Reprod Sci.

[3] Vidal A, Wachter C, Kohl Schwartz A, Dhakal C (2021). A rare presentation of isolated right-sided pleural effusion in the context of ovarian hyperstimulation syndrome: A case report. Case Rep Womens Health.

[4] Taniguchi LU, Jorge CG, de Oliveira LF (2011). Spontaneous bacterial peritonitis complicating ovarian hyperstimulation syndrome-related ascites. Clinics (Sao Paulo).

[5] Mullin CM, Fino ME, Reh A, Grifo JA, Licciardi F (2011). Symptomatic isolated pleural effusion as an atypical presentation of ovarian hyperstimulation syndrome. Case Rep Obstet Gynecol.

[6] Khalil MA, Ghazni MS, Tan J, Naseer N, Khalil MA (2016). Spontaneous bacterial peritonitis and anasarca in a female patient with ovarian hyperstimulation syndrome complicated by respiratory and kidney failure. Case Rep Gastroenterol.

[7] Kwik M, Maxwell E (2016). Pathophysiology, treatment and prevention of ovarian hyperstimulation syndrome. Curr Opin Obstet Gynecol.

[8] Henshaw CA, Kirschen GW, Chen L, Vaught AJ, Cameron K, Christianson M (2022). Severe ovarian hyperstimulation syndrome requiring recurrent large-volume paracenteses until 21 weeks' gestation: a case report. F S Rep.

[9] Yildizhan R, Adali E, Kolusari A, Kurdoglu M, Ozgokce C, Adali F (2008). Ovarian hyperstimulation syndrome with pleural effusion: a case report. Cases J.

[10] (2008). Ovarian hyperstimulation syndrome. Fertil Steril.

[11] Ovarian Stimulation TE, Bosch E, Broer S (2020). ESHRE guideline: ovarian stimulation for IVF/ICSI(†). Hum Reprod Open.

[12] Namavar Jahromi B MD, Parsanezhad ME MD, Shomali Z MD, Bakhshai P MD, Alborzi M MD, Moin Vaziri N MD PhD, Anvar Z PhD (2018). Ovarian hyperstimulation syndrome: a narrative review of its pathophysiology, risk factors, prevention, classification, and management. Iran J Med Sci.

